# Enrolment of children in psychosocial care: problems upon entry, care received, and outcomes achieved

**DOI:** 10.1007/s00787-017-1048-1

**Published:** 2017-11-08

**Authors:** Marieke Nanninga, Danielle E. M. C. Jansen, Erik J. Knorth, Sijmen A. Reijneveld

**Affiliations:** 1 Department of Health Sciences, University Medical Center Groningen, University of Groningen, Antonius Deusinglaan 1/FA10, 9713 AV Groningen, The Netherlands; 2 Department of Sociology and Interuniversity Center for Social Science Theory and Methodology (ICS), University of Groningen, Groningen, The Netherlands; 3 Department of Special Needs Education and Youth Care, University of Groningen, Groningen, The Netherlands

**Keywords:** Child, Adolescent, Psychosocial care, Treatment outcomes, Health services accessibility

## Abstract

Psychosocial care systems have been designed so that specific problems are treated by specific care types. There is insufficient evidence as to which problem types are actually presented to the various care types. This study assessed types and severity of problems among children and adolescents upon enrolment in psychosocial care, compared to children not enrolled; also outcomes after 3 and 12 months, overall and per care type. We obtained data on a cohort of 1382 Dutch children aged 4–18 years (response rate 56.6%), included upon enrolment in psychosocial care, and on 443 not-enrolled children (response rate 70.3%), all from one region. Results showed that enrolled children had more problems than children not enrolled in care. In child and adolescent mental healthcare (CAMH), relatively many children had internalizing problems, and in child and adolescent social care (CASC) relatively many children had externalizing, parenting, family and multiple problems. Regardless of the type of problem, care duration in preventive child healthcare (PCH) was relatively short; and in CASC and CAMH longer. After 3 and 12 months, rates of problem solution were highest in PCH. These rates were also substantial among children not in care. To conclude, our findings show that the system of psychosocial care functions as intended regarding the distribution of problems across care types. Extended demarcation of clients by problem type and severity towards type and contents of care may further improve the system.

## Introduction

Children and their families enrol in psychosocial care because of various child and family problems [1, 2]. However, evidence is lacking as to which types of problems are presented to which types of psychosocial care, even though the idea behind the system of psychosocial care would seem self-evident [3]. Evidence is also lacking as to whether care outcomes vary depending on the alignment of type of problems with type of psychosocial care. Such knowledge is needed to optimize the access of children and adolescents to psychosocial care, to deliver care that is need-oriented, and to improve care outcomes [4–7].

Systems for psychosocial care have been designed so that specific care types focus on specific child and family problems, depending on levels of problem severity and co-occurrence of problems in the social and/or economic context of the child [8–10]. For example, in The Netherlands, preventive child healthcare (PCH) focuses on mild child and family problems, referring children and families with more severe problems. Specialized child and adolescent mental healthcare (CAMH) provides care for children with more severe psychosocial problems and psychiatric disorders. Child and adolescent social care (CASC), in addition to dealing with children’s psychosocial problems, focuses on problems in the social and economic context that could impede or threaten the child’s development, problems such as poor parenting and unhealthy family functioning [3, 8].

Types of psychosocial care have thus been delineated by design, but there is insufficient evidence as to whether this delineation is realized in practice. The few available studies suggest at least some overlap in the types of problems addressed by the various care types. However, these previous studies have addressed only the child’s problems, without considering problems in their social and economic context [3, 11–14]. Moreover, there is little evidence on the effectiveness of the design itself: which problems could best be referred to which types of psychosocial care.

The aim of our study was thus to assess the types and severity of problems upon enrolment in psychosocial care, i.e. child, parenting and family problems, and compare these to problems of children and adolescents not enrolled in psychosocial care. Next, we assessed outcomes, i.e. care duration and problem solution, after 3 and 12 months.

## Methods

### Study design

We used one-year follow-up data from TakeCare, a large prospective cohort study of children aged 4–18 in the northeast of The Netherlands [8]. TakeCare has been designed to investigate the trajectories and outcomes of children receiving psychosocial care and consists of a care cohort of children enrolling in psychosocial care, and a reference cohort of children not in care. Between April 2011 and April 2013, parents/caregivers of children aged 4–18 years, along with children aged 12 years and over, were invited to participate in TakeCare. The Medical Ethical Committee of the University Medical Center Groningen evaluated the design of the study, and approved it without requiring full assessment. Informed consent was obtained from all participating respondents [8].

### Sample and procedure

For the care cohort, 2664 children and their parents/caregivers were recruited via PCH, CASC and CAMH, the main providers of psychosocial care for children and adolescents in The Netherlands. At the time of our study, children entered psychosocial care via either their general practitioner, the youth care office, or PCH [15]. General practitioners and doctors and nurses in PCH provide light psychosocial support to children and their families. In case of more severe problems they refer children to specialized care, either to CASC, primarily staffed by child (social) workers, or to CAMH, primarily staffed by child psychologists and psychiatrists [3]. See Table 1 for a detailed description of the care types.

**Table 1:** The main providers of psychosocial care for children and adolescents in The Netherlands; the situation at the time of the data collection

Care type	Description
Preventive child healthcare (PCH)	In PCH, doctors and nurses provide care to children and families with mild child and family problems. In case of more severe problems, PCH may refer children and families to specialized care, either CASC or CAMH PCH provides light psychosocial support, for example family support on an ambulatory/outpatient or home-based basis. Care aims to be short Children and families mainly enter PCH by visiting the school doctor or nurse who is employed in PCH. Enrolment via referral of their general practitioner or via the youth care office (in Dutch: ‘Bureaus Jeugdzorg’) is also possible Municipalities finance PCH
Child and adolescent social care (CASC)	In CASC, child (social) workers and pedagogues provide specialized care to children and families. CASC treats psychosocial problems and problems in the social and economic context that impede or might threaten the child’s development, such as parental or family problems. Compared to PCH, CASC treats more severe problems CASC provides individual child support, trauma support, experiential learning support, independent living support, parenting and family support and foster care support. Care includes ambulatory/outpatient, home-based, day treatment, residential care or family foster care. More frequently than in PCH, care lasts longer than 3 months Children and families enter CASC mainly via referral by the youth care office. The youth care office also decides about the type of interventions that needs to be offered. Referral to CASC by PCH or the general practitioner is also possible Provincial governments finance CASC
Child and adolescent mental healthcare (CAMH)	In CAMH psychologists and psychiatrists provide specialized care to children and families. CAMH treats psychosocial problems and psychiatric disorders. Compared to PCH, CAMH treats more severe problems CAMH provides individual child support, trauma support, parenting and family support. Care is ambulatory/outpatient, home-based or day-treatment. More frequently than in PCH, care lasts longer than 3 months Children and families enter CAMH mainly via referral by the general practitioner. Referral via PCH or the youth care offices is also possible Health insurance companies finance CAMH

Since the new Child and Youth Act became operational in 2015 municipalities are responsible for all three care types. This table is based on Evenboer [16]; Reijneveld et al. (2014) [3]; Verhage et al. (2014) [8]

Children with insufficient understanding of Dutch, living outside the northern region, or following special education because of intellectual disability, were excluded (*N* = 223). Of the eligible (either child and/or parent) 2441 respondents, 1382 participated (response 56.6%). Differences between respondents and non-respondents were small regarding characteristics known to influence enrolment and outcomes and available for non-respondents: age, gender, degree of urbanization (i.e. rural versus urban based on density of living addresses per zip code), and psychosocial problems. For these Cohen’s effect sizes ranging from 0.01 (age) to 0.12 (degree of urbanization) [8, 17, 18].

For the reference cohort a stratified random sample of 1025 school children and their parents was approached. The sample was obtained via five primary schools, two secondary schools, and one school for intermediate vocational education. Thereby the distribution of children across the study region according to age, gender, socioeconomic position, and degree of urbanization was taken into account. Of these children, 77 were excluded using the same exclusion criteria as with the care cohort. Of the eligible 948 respondents, 666 participated (70.3%). Differences between respondents and non-respondents were small regarding age, gender, degree of urbanization, and psychosocial problems, with effect sizes ranging from 0.02 (psychosocial problems) to 0.08 (degree of urbanization) [8]. Children who had had contact with psychosocial care in the past 6 months were excluded. This resulted in a reference cohort of 443 participants.

Data were obtained from parents/caregivers and adolescents via web-based or paper questionnaires at three moments. If required, we provided assistance in filling out the questionnaire. The baseline measurement (T1) followed directly after entry into the study, which was, for the care cohort, at the moment of the child’s enrolment. The second (T2) and third (T3) questionnaires were sent 3 and 12 months after the first questionnaire, respectively. The loss to follow-up at T2 and T3 was 6.9 and 8.8% for the care cohort and 2.0 and 2.2% for the reference cohort, respectively [8].

### Measures

*Types of problems *concerned child, parenting and family problems upon entry into the study (T1), after three months (T2) and after one year (T3). Child problems concerned internalizing and externalizing problems measured using the Strengths and Difficulties Questionnaire (SDQ) [19–21]. The SDQ consists of 25 items describing positive and negative attributes of children with regard to emotional problems, behavioral problems, hyperactivity, peer problems, and prosocial behavior. We measured internalizing problems as the sum of ten items related to emotional and peer problems [Cronbach’s *α* parents = 0.78 (T1, T2, T3), adolescents = 0.75 (T1), 0.76 (T2), 0.73 (T3)], and externalizing problems as the sum of ten items related to behavioral problems and hyperactivity [Cronbach’s *α* parents = 0.83 (T1, T2, T3), adolescents = 0.74 (T1), 0.76 (T2), 0.73 (T3)] [22]. Scores were (a) dichotomized as ‘problems’ if either the parent score or the adolescent score was increased, versus ‘no problems’, and (b) counted as change in mean severity (ranges 0–20).

*Parenting problems *were measured using total parent scores on the nine-item version of the Alabama Parenting Questionnaire (APQ) [23]. The APQ includes a five-point Likert scale on the domains poor supervision, inconsistent disciplining and positive parenting (maximum of three missing items, Cronbach’s *α* = 0.66 (T1, T2), 0.69 (T3)). The total APQ scale was dichotomized into ‘problems’ (score 2.25–5), based on the 20% highest scores in the total reference cohort on T1 and otherwise into ‘no problems’, i.e. reflecting the SDQ cut-off points. Scores were also summed per measurement, leading to changes in severity between measurements (range 1–5).

*Family problems *were measured using the General Functioning Scale (GF) of the McMaster Family Assessment Device (FAD) [24, 25]. Using 12 items, the scale included the dimensions problem solving, communication, roles, affective responsiveness and involvement, and behavioural control. Parents rated their agreement on a four-point scale from ‘totally disagree’ to ‘totally agree’ (maximum of two missing items, Cronbach’s *α* = 0.87 (T1, T2), 0.83 (T3)). The GF was (a) dichotomized into ‘problems’, i.e. unhealthy family functioning, or ‘no problems’, i.e. healthy family functioning, and (b) counted as change in mean severity (range 1–4) [26].

*Number of problems* was measured by combining the dichotomized scores on internalizing, externalizing, parenting and family problems, resulting in five categories ranging from problems in no domain to problems in four domains. For example, a child with both internalizing and externalizing problems, and a child with both internalizing problems and parents who had parenting problems fell in the second category.

*Types of psychosocial care *referred to the psychosocial care service by which children entered this study, categorized as ‘PCH’, ‘CASC’ or ‘CAMH’. Children in the reference cohort were categorized as ‘Not in care’.

*Duration of care* as a process-outcome was included and defined as (0) ‘0–3 months’, (1) ‘> 3 months-1 year’, and (2) ‘> year’, based on information from adolescents, and from the parents if no child information was available.

*Outcomes after three and twelve months* referred to problem solution with *resolved problems *and *change in severity *per type of problem (internalizing, externalizing, parenting and family problems). *Resolved problems *referred to a change from a ‘problem’ score at T1 to a ‘no problems’ score at T2 and T3, respectively. *Change in problem severity *referred to a change between T1, T2 and T3, respectively.

*Background characteristics* included age, gender, ethnicity, psychosocial care use in the past six months, parental educational level, and family composition (T1). *Age* was categorized as 4–11, and 12–19, as in the Dutch educational system the primary school age includes ages 4–11; and secondary school age is from 12 years onwards; the child psychosocial care system is focused on children up to 18 years old. *Ethnicity* was defined as either Dutch or non-Dutch (the child and/or one of the parents was foreign-born). *Psychosocial care use in the past six months *was measured with the Questionnaire Intensive Care for Youth (QUINCY) [27–30]. Parents and adolescents reported whether they had used care because of the child’s psychosocial problems, and if so, which type(s) and by which professional. *Past use of care *was defined as the use of professional care for psychosocial problems of the child during the past 6 months.

*Parental educational level *was based on the highest educational level achieved by either one of the parents/caregivers [31]. *Family composition *was assessed by asking the parent and the adolescent with whom the child lived. This was categorized into ‘biological two-parent family’ and ‘other’ (e.g., living with one parent, a foster family or living in a residential care facility).

### Analyses

First, we described the characteristics of the cohorts. Second, we assessed the types of problems of children being enrolled, as compared to children not enrolled, in psychosocial care, per type of care enrolled in. Third, we assessed the duration of care and problem solution (removal and reduction of severity of problems) between the types of care, per type of problem. We performed the analyses on the reduction of severity of problems using Generalized Linear Mixed Modelling in SAS (http://www.sas.com), taking into account the hierarchical nature of the pre-post data. We repeated all analyses after the exclusion of those receiving psychosocial care before T2 or T3 from the group of children and adolescents not enrolled in care.

## Results

### Participants’ characteristics

The majority of the enrolled children were 4–11 years old; a slight majority were male and living in other than a biological two-parent family. Among children aged 12–19 years, parents reported psychosocial problems more frequently than did children. Of the children not enrolled in care, the majority were also 4–11 years of age; a large majority were female and lived with their biological parents. Contrary to the enrolled group, children aged 12–19 years in the non-enrolled group reported problems more frequently than did parents (Table 2). For children aged 4–11 years, no child report was available due to their young age.

**Table 2:** Characteristics of the participating children and adolescents aged 4–19 years by enrolment status (enrolled or not enrolled in psychosocial care)

Characteristics	Enrolled in psychosocial care (care cohort)		Not enrolled in psychosocial care (reference cohort)	
	*N* = 1382^a^		*N* = 443^a^	
	*N*	(%)	*N*	(%)
Child characteristics				
Age				
4–11 years (i.e. primary school age)	828	(60.1)	268	(60.5)
12–19 years (i.e. secondary school age and older)	550	(39.9)	175	(39.5)
Gender				
Male	734	(53.3)	184	(41.5)
Female	644	(46.7)	259	(58.5)
Ethnicity				
Dutch	1098	(84.6)	395	(93.4)
Non-Dutch	200	(15.4)	28	(6.6)
Internalizing problems (parent report)				
Normal	468	(35.2)	384	(88.9)
Borderline/abnormal	863	(64.8)	48	(11.1)
Internalizing problems (adolescent report)				
Normal	227	(50.1)	135	(82.3)
Borderline/abnormal	226	(49.9)	29	(17.7)
Externalizing problems (parent report)				
Normal	675	(50.7)	413	(95.6)
Borderline/abnormal	656	(49.3)	19	(4.4)
Externalizing problems (adolescent report)				
Normal	263	(58.1)	143	(87.2)
Borderline/abnormal	190	(41.9)	21	(12.8)
Psychosocial care use in past six months				
No	224	(16.3)	443	(100.0)
Yes	1154	(83.7)	0	(0.0)
Parent and family characteristics				
Parental educational level				
Low	242	(17.6)	32	(7.4)
Medium	694	(50.4)	207	(47.9)
High	387	(28.4)	193	(44.7)
Family composition				
Biological two-parent family	652	(47.3)	328	(74.0)
Other	723	(52.6)	115	(26.0)
Parenting problems				
No	949	(71.5)	373	(86.3)
Yes	378	(28.5)	59	(13.7)
Family problems				
No	886	(66.8)	398	(92.3)
Yes	440	(33.2)	33	(7.7)
Care-related characteristics				
Type of psychosocial care				
Preventive child healthcare	366	(26.6)	–	–
Child and adolescent social care	234	(17.0)	–	–
Child and adolescent mental healthcare	778	(56.5)	–	–
Not in care	–	–	443	(100.0)

^a^ Numbers do not always add up to *N* = 1382 and *N* = 433 due to missing data

The distribution of age and gender over the three care types showed that children enrolled in PCH were mainly 4–11 years old with an even distribution for gender. In CASC more children were 12–19 years old and female, and in CAMH more were 4–11 years old and male (Table 3).

**Table 3:** Children and adolescents aged 4–19 years, enrolled versus not enrolled in care: type and number of problems, and type of care enrolled in

Age and gender	Enrolled in care		Not enrolled in care		P^1^	PCH		CASC		CAMH		P^2^
	*N* = 1378		*N* = 443			*N* = 366		*N* = 234		*N* = 778		
	*N*	(%)	*N*	(%)		*N*	(%)	*N*	(%)	*N*	(%)	
**Age**												
4–11 years (i.e. primary school age)	828	(60.1)	268	(60.5)		316	(86.3)	100	(42.7)	412	(53.0)	***
12–19 years (i.e. secondary school age and further)	550	(39.9)	175	(39.5)		50	(13.7)	134	(57.3)	366	(47.0)	
**Gender**												
Male	734	(53.3)	184	(41.5)	***	185	(50.5)	102	(43.6)	447	(57.5)	***
Female	644	(46.7)	259	(58.5)		181	(49.5)	132	(56.4)	331	(42.5)	
**Problems T1**												
Internalizing problems	916	(66.5)	70	(15.8)	***	202	(55.2)	148	(63.2)	566	(72.8)	***
Externalizing problems	739	(53.6)	39	(8.8)	***	143	(39.1)	146	(62.4)	450	(57.8)	***
Parenting problems	378	(28.5)	59	(13.7)	***	73	(20.2)	80	(38.5)	225	(29.7)	***
Family problems	440	(33.2)	33	(7.7)	***	92	(25.4)	97	(46.6)	251	(33.2)	***
Number of problems												
No problems	172	(13.0)	282	(65.4)	***	93	(25.8)	19	(9.2)	60	(7.9)	***
1 problem	367	(27.7)	108	(25.1)		109	(30.2)	41	(19.8)	217	(28.7)	
2 problems	424	(32.0)	35	(8.1)		97	(26.9)	63	(30.4)	264	(35.0)	
3 problems	231	(17.5)	3	(0.7)		44	(12.2)	51	(24.6)	136	(18.0)	
4 problems	129	(9.8)	3	(0.7)		18	(5.0)	33	(15.9)	78	(10.3)	

*PCH* preventive child healthcare, *CASC* child and adolescent social care, *CAMH* child and adolescent mental healthcare

P^1^: *p* value for differences between the enrolled and non-enrolled group; P^2^: *p* value for differences by type of care, for the enrolled group; ^#^ *p* < 0.10, * *p* < 0.05, ** *p* < 0.01, *** *p* < 0.001

### Types of problems upon enrolment

Children enrolled in psychosocial care most often had internalizing problems, followed by externalizing problems, family problems and parents with parenting problems (Table 3). They usually had one or two problems. Children not enrolled in psychosocial care usually had no child, parenting or family problems. If they had a problem, it was most frequently only one problem, and involved internalizing problems or parenting problems. One problem mostly concerned internalizing problems (not in care: 38.0%; enrolled in care: 58.3%). Two problems mainly concerned internalizing and externalizing problems (not in care: 28.6%; enrolled in care: 58.0%). Three problems mainly concerned internalizing and externalizing with parenting problems (not in care: 66.7%) or with family problems (enrolled in care: 25.5%) (data not shown).

Regarding the link between type of problems and type of care enrolled in, internalizing problems occurred relatively most frequently in CAMH. Externalizing problems, parenting problems and family problems occurred relatively most frequently in CASC. Regarding the number of problems, three or four problems occurred relatively most frequently in CASC and two problems most frequently in CAMH. Finally, no problems or only one problem occurred most frequently in PCH.

### Outcomes

Results on outcomes showed that for children and adolescents with any problem upon enrolment, care duration was short in PCH and longer in CASC and CAMH (Table 4). Regarding the other outcomes, children not in care as well as children in PCH showed the highest frequency of resolved problems, i.e. no longer having any problem, both after three and twelve months.

**Table 4:** Outcomes of children and adolescents aged 4–19 years after three and twelve months, i.e. care duration and problem solution

Type of problems (T1) per care type or not in care	Enrolment				Process-outcome			Outcomes after 3 months		Outcomes after 12 months	
	Problems^a^	Problem severity			Care duration			Resolved problems^bc^	Problem severity change^b^	Resolved problems^bc^	Problem severity change^b^
					0–3 months	> 3–12 months	> 12 months				
	*N*	*M*	(SE)		%	%	%	Δ (%)	Δ (%)	Δ (%)	Δ (%)
Internalizing problems											
PCH	202	9.0	(0.19)***		56.8	13.6	29.5***	−38.1**	−20.5**	−54.0***	−34.2***
CASC	148	8.9	(0.21)		32.5	23.0	44.4	−26.4	−10.6	−39.2	−15.8
CAMH	556	9.4	(0.12)		34.5	23.3	42.1	−26.3	−16.4	−34.6	−23.4
Not in care	70	6.8	(0.24)		–	–	–	−40.0	−13.3	−54.3	−22.3
Externalizing problems											
PCH	143	11.4	(0.21) ***		46.3	18.7	35.0^#^	−33.6	−12.7	−48.3	−21.7^#^
CASC	146	11.4	(0.26)		32.8	25.6	41.6	−34.2	−11.5	−49.3	−22.7
CAMH	450	11.3	(0.12)		36.4	18.6	45.1	−30.2	−11.7	−42.9	−17.8
Not in care	39	8.8	(0.32)		–	–	–	−43.6	−10.3	−53.8	−13.7
Parenting problems											
PCH	73	2.6	(0.03) ***		63.3	20.0	16.7*	−54.8^#^	−11.6*	−61.7^#^	−15.8**
CASC	80	2.7	(0.04)		37.1	21.4	41.4	−36.6	−9.5	−38.5	−10.4
CAMH	225	2.6	(0.02)		36.0	23.4	40.7	−38.3	−7.0	−44.5	−9.5
Not in care	59	2.5	(0.03)		–	–	–	−35.6	−5.6	−48.3	−8.0
Family problems											
PCH	92	2.3	(0.03)**		50.7	20.5	28.8^#^	−39.7^#^	−7.9	−44.6	−12.5
CASC	97	2.5	(0.04)		41.9	17.4	40.7	−35.6	−9.4	−40.5	−11.0
CAMH	251	2.4	(0.02)		34.1	23.7	42.2	−38.2	−7.7	−42.3	−11.9
Not in care	33	2.2	(0.04)		–	–	–	−60.6	−16.9	−53.1	−19.3
Any problem											
PCH	268	1.9^d^		(0.05)***	56.0	15.8	28.2***	−20.0^e^***	–	−31.1^e^***	–
CASC	188	2.4^d^		(0.07)	37.2	22.1	40.7	−8.9^e^	–	−16.7^e^	–
CAMH	695	2.1^d^		(0.04)	37.3	22.8	40.0	−11.9^e^	–	−17.9^e^	–
Not in care	149	1.3^d^		(0.05)	–	–	–	−27.8^e^	–	−42.7^e^	–

*PCH* preventive child healthcare, *CASC* child and adolescent social care, *CAMH* child and adolescent mental healthcare

^a^ This refers to the number of children between types of care per type of problem as reported in Table 3

^b^ This refers to the type of problems upon enrolment as indicated in the first column

^c^ The minus-figure the decrease in prevalence of problems

^d^ This refers to the average number of problems

^e‘^Resolved problems’ regards the percentage of children and adolescents that had at least one problem at T1 (ranging between 1 and 4 problems) minus the percentage of all children that had at least one problem at T2 (or T3)

^#^ *p* < 0.10, * *p* < 0.05, ** *p* < 0.01, *** *p* < 0.001

Results per type of problems upon enrolment showed that for internalizing problems, care duration was also short in PCH and longer in CASC and CAMH. Internalizing problems were most frequently resolved among children not in care and in PCH, both after three and twelve months. Decrease in severity of internalizing problems was highest in PCH. Regarding externalizing problems upon enrolment, no significant differences occurred between the care types or between those enrolled or not enrolled in care.

For parenting problems, care duration was also shortest in PCH and longer in CASC and CAMH. No significant differences emerged between the four groups in the frequency of parenting problems resolved. Decrease of severity of parenting problems was highest in PCH, both after three and twelve months. Finally, regarding family problems, no significant differences emerged between groups.

Exclusion of children and adolescents receiving psychosocial care before T2 or T3 from the group not enrolled in care (*n* = 130) yielded somewhat greater differences between the children enrolled in care and the group not enrolled in care, but without affecting the general pattern (not shown).

## Discussion

In general our findings confirm the principles behind the system of psychosocial care for children and adolescents. Children enrolled in PCH had mild problems compared to children in CASC and CAMH. In CAMH, relatively many children had internalizing problems, and in CASC relatively many children had externalizing, parenting, family and multiple problems (child problems and problems related to the child’s context, respectively). Further, care duration was relatively short in PCH and longer in CASC and CAMH. Finally, problems were resolved most often in PCH and among children not in care.

These findings confirming the principles of the system are in line with the limited previous findings. First, PCH treats mild problems, and CASC and CAMH more severe problems. This may explain the short care duration and greater problem solution in PCH compared to CASC and CAMH [32, 33]. More severe problems—most likely also more complex, more persistent and combined with other problems—can be expected to be more difficult to treat and, therefore, to require longer treatment [32, 33]. Second, our study shows that, compared to CAMH, CASC focuses more on the social and/or economic context of the child, as well as on the child’s problems. This confirms, for example, the available evidence that parental divorce has a stronger association with the use of CASC than with the use of CAMH [3]. The system thus seems to perform as intended regarding the distribution of problems across care types. Third, children in PCH are much younger than in the other types of care, due to the focus of PCH on primary schools. This younger age may contribute to differences in outcomes.

Our study showed that some children enrolled in care did not seem to have problems—13%, and that some children not enrolled in care did seem to have problems—35%; this confirms previous findings [2, 34–40]. The first observation—children enrolled ‘without problems’—might imply that other reasons for enrolment occurred, in particular the existence of a threat of developing problems or other care needs, as in cases of parental divorce with strong conflicts or previous hospitalization [5, 32, 36]. It is also possible that problems which existed initially were resolved rather quickly, before the SDQ was scored [39]. Finally, the observation might also simply indicate overtreatment [2, 34–40]. In any case, the first explanation holds. The second observation—children with problems not enrolled in care—may imply undertreatment caused by barriers to access to care, barriers involving problem recognition, help seeking or referral [30, 41–45]. It might be that some of these barriers are resolved later on. This is supported by our finding that 29% of the children not enrolled initially contacted a professional, usually the general practitioner, for light psychosocial support after 3 or after 12 months. An explanation may be that problems are not recognized by the parents. The finding that adolescents in the non-enrolled group reported higher problem levels than their parents, and that the contrary holds for enrolled adolescents, somewhat supports this explanation. It may also be that children with problems not enrolled in care consider themselves able to cope with their problems, or do not really consider them problematic [35, 36, 46, 47]. Further research is needed to disentangle these explanations.

Regarding outcomes, we found that the decrease in problem severity was relatively strong for clients enrolled with internalizing or parenting problems in PCH compared to those enrolled in CASC and CAMH. An explanation for this may be that problems in PCH less often have other concomitant psychosocial problems, compared to CASC and CAMH. Especially in CASC, problems are often multiple, i.e. almost 40% of the children enrolled in CASC had three or four problems. Singular problems are more likely to be easily changed than are multiple problems. Resolving problems may, therefore, take more time in CASC and CAMH. These findings on outcomes also confirm that the system works as intended: light and short care for mild and singular problems that are easily resolved, and specialized and longer care for severe and concomitant problems that are more persistent [48, 49].

Between clients of the three care types and those not enrolled in care with externalizing or family problems, our study showed a substantial overlap in outcomes. In addition, for clients with internalizing or parenting problems, outcomes were rather similar in CASC and in CAMH. This implies that the system as designed and realized does not highly affect problem solution; e.g. child context problems are not resolved more often in CASC than in CAMH. As far as we know this is the first study to compare several outcomes between various care types with the problem type upon enrolment. Further research is needed, for example, on the types of interventions offered within each care type, to determine which type of care best applies to which type of problem [9, 10].

Regarding problem solution, we found substantial but by far not complete reductions in problem rates, i.e. 35–62% after 12 months, confirming previous research [1, 5, 50, 51]. An explanation might be that treatment is not always aimed at problem solution, but sometimes just at making problems more manageable, as not all disorders can be cured [52]. We also found substantial problem reduction among children not in care—e.g. 43% for any problem after 12 months. This again confirms earlier findings [53, 54], but with more robust data. Our finding suggests that problems among children and adolescents not enrolled in care resolve spontaneously more easily because they are less severe and usually not accompanied by other psychosocial problems. In addition, our study showed that children not enrolled in care live in a more favourable context, such as with their two biological parents, with parents with relatively higher educational levels, and more often off Dutch origin (for comparable findings, see [41, 55, 56]). These findings on outcomes might also be seen to suggest that treatment is, on average, only to some extent effective, with some children and/or parents reaping more benefit than others [1, 50]. Insight into the impact of care on other outcomes, such as coping with problems, could lead to a better understanding of this issue.

### Strengths and limitations

This study has considerable strengths. First, we were able to make longitudinal comparisons between children and adolescents enrolled in care and those not enrolled, with high retention and in a large sample. Second, we were able to include all children and adolescents in a well-defined catchment area, providing an inclusive overview of all types of psychosocial care.

Our study also had some limitations. First, we had a considerable non-response upon entry. However, differences between respondents and non-respondents were small, decreasing the likelihood of selection bias [8]. Second, the observational nature of this study limits its potential causal inferences on outcomes of care. Third, although we included both parents and adolescents reported data, we did not have adolescents’ reports for some problem domains, such as for parenting and family problems.

### Implications

Our study provides a first sketch of the association between problems upon enrolment, care types, and outcomes. Essentially, we found that the system of psychosocial care seems to perform as intended regarding the distribution of problems across care types. Our findings also suggest several starting points for the improvement of the system, e.g. regarding (1) children enrolled in care without problems and children not enrolled in care with problems, (2) overlap in outcomes between care types, and (3) only partial solution of problems.

The first issue has to do with improving the process of enrolment in care. We particularly need to disentangle the reasons why some children without problems enrol in care, and others with problems do not, to show whether or not this is a desired situation. A related issue is to further disentangle why adolescents in the non-enrolled group scored higher on psychosocial problems compared to their parents, and why this was the opposite in the enrolled group, and whether this difference is related to the process of enrolment in care.

The second issue, overlap in outcomes, calls for further research on the specific interventions offered in each type of care to assess whether or not the type of care makes any difference. For example, internalizing problems caused by trauma probably require other interventions than those caused by phobia [57]. Such research may also indicate to what extent care types are (dis)similar and whether the intentions of the system should become more specific and demarcated (i.e., CAMH specializes in the child’s problems; and CASC specializes in the child’s context). In addition, further research should explain to what extent the solution of one problem type, affects the solution of another problem type.

The third issue shows a need for further research into the impact of psychosocial care on other outcomes, such as coping strategies, or impairments in societal participation of children and their caretakers [57]. This could also include an assessment of underlying characteristics affecting prognosis, in multivariate analyses. Such research could further improve our understanding of the role and importance of psychosocial care for children and their families.

Finally, our findings need confirmation by and comparison with other systems of psychosocial care for children. Apparently such systems vary, though comparative research throughout the European Union shows a rather striking resemblance across countries [58]. This suggests a major global change in improving care for children and adolescents [2].
